# Supplementary material for: Western Diet Modifies Platelet Activation Profiles in Male Mice
Source: Int J Mol Sci. 2024 Jul 23;25(15):8019. doi: 10.3390/ijms25158019 (PMC11311362; doi:10.3390/ijms25158019)
Supplement: Supplementary file 1 [file ijms-25-08019-s001.zip › ijms-3073117-supplementary.pdf]

International Journal of
Molecular Sciences

MDPI

Supplementary Materials

Western Diet Modifies Platelet Activation Profiles in Male Mice

Adam Corken, Elizabeth C. Wahl, James D. Sikes and Keshari M. Thakali

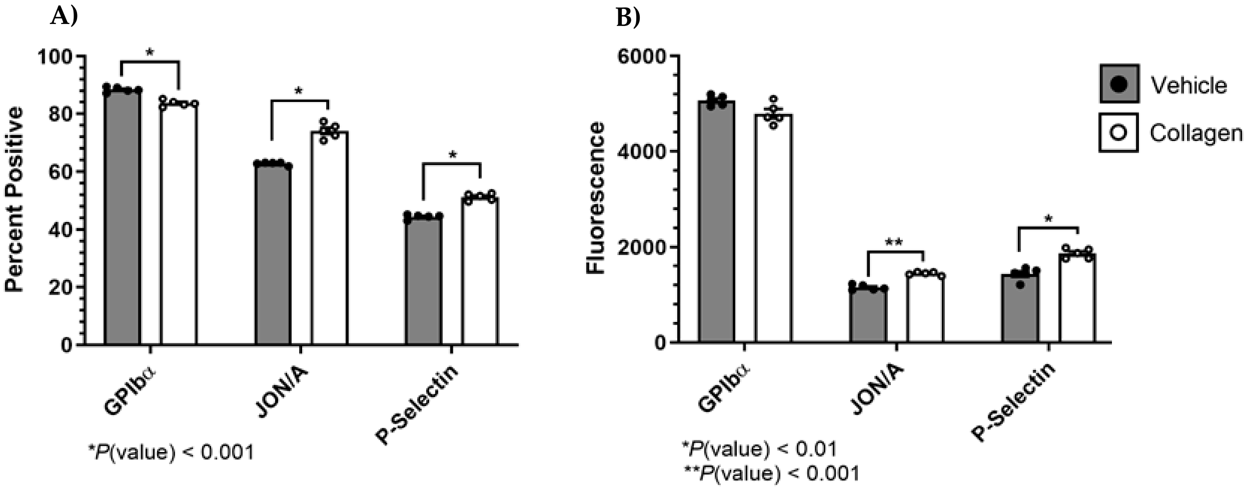

Supplementary Figure S1. A) and B) Validation of collagen induced platelet stimulation. PRP isolated from male mice fed a chow diet was utilized as a preliminary means of determining collagen induced changes in surface receptor expression. All parameters assessed yielded an observable change in control (vehicle) relative to collagen treated samples with the exception of GPIbα fluorescence.

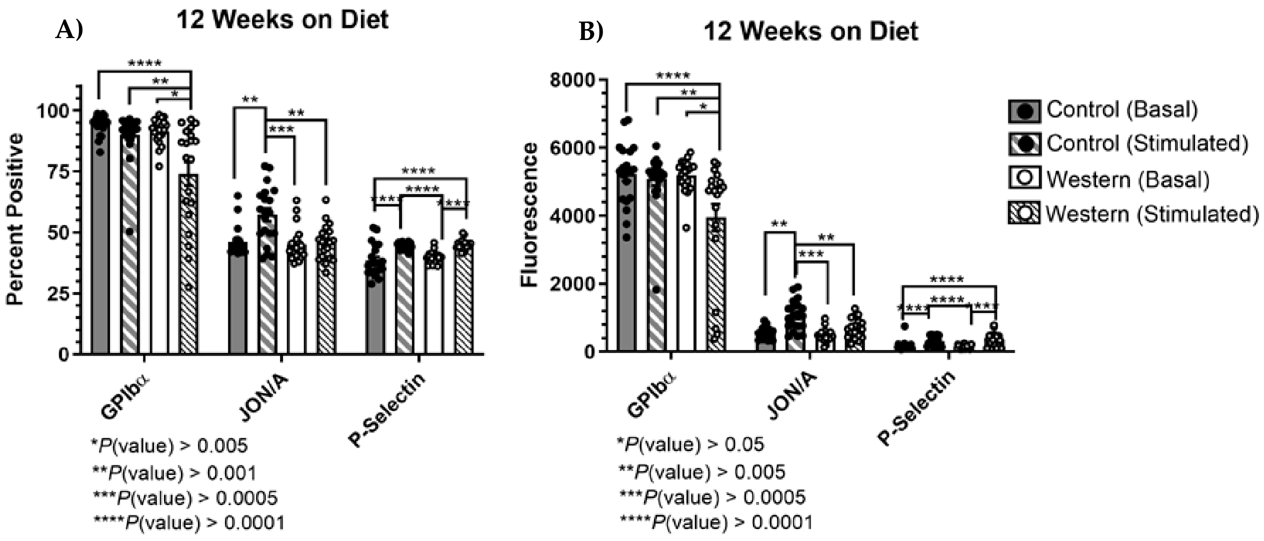

Supplementary Figure S2. A) and B) The comparison of basal levels of platelet receptor expression to collagen stimulated samples was undertaken at the 12 week time point to validate the effectiveness of collagen stimulation. Control samples were comparable to our preliminary validation findings with JON/A and P-selectin levels increasing with collagen stimulation and GPIbα levels remaining unchanged. Interestingly, it appears a Western diet primes platelets to exhibit a reduction in GPIbα levels while also diminishing the increase in JON/A staining following collagen stimulation.
